# Optimizing classification of diseases through language model analysis of symptoms

**DOI:** 10.1038/s41598-024-51615-5

**Published:** 2024-01-17

**Authors:** Esraa Hassan, Tarek Abd El-Hafeez, Mahmoud Y. Shams

**Affiliations:** 1 Faculty of Artificial Intelligence, Kafrelsheikh University, Kafrelsheikh, 33516 Egypt; 2 Department of Computer Science, Faculty of Science, Minia University, Minia, 61519 Egypt; 3 Computer Science Unit, Deraya University, Minia University, Minia, 61765 Egypt

**Keywords:** Diseases, Health care, Computer science, Information technology, Scientific data

## Abstract

This paper investigated the use of language models and deep learning techniques for automating disease prediction from symptoms. Specifically, we explored the use of two Medical Concept Normalization—Bidirectional Encoder Representations from Transformers (MCN-BERT) models and a Bidirectional Long Short-Term Memory (BiLSTM) model, each optimized with a different hyperparameter optimization method, to predict diseases from symptom descriptions. In this paper, we utilized two distinct dataset called Dataset-1, and Dataset-2. Dataset-1 consists of 1,200 data points, with each point representing a unique combination of disease labels and symptom descriptions. While, Dataset-2 is designed to identify Adverse Drug Reactions (ADRs) from Twitter data, comprising 23,516 rows categorized as ADR (1) or Non-ADR (0) tweets. The results indicate that the MCN-BERT model optimized with AdamP achieved 99.58% accuracy for Dataset-1 and 96.15% accuracy for Dataset-2. The MCN-BERT model optimized with AdamW performed well with 98.33% accuracy for Dataset-1 and 95.15% for Dataset-2, while the BiLSTM model optimized with Hyperopt achieved 97.08% accuracy for Dataset-1 and 94.15% for Dataset-2. Our findings suggest that language models and deep learning techniques have promise for supporting earlier detection and more prompt treatment of diseases, as well as expanding remote diagnostic capabilities. The MCN-BERT and BiLSTM models demonstrated robust performance in accurately predicting diseases from symptoms, indicating the potential for further related research.

## Introduction

In the field of healthcare, accurate and timely diagnosis of diseases is of paramount importance for effective treatment and patient care^1–3^. Traditionally, medical professionals rely on their expertise and diagnostic tests to identify diseases based on a patient's symptoms. However, this process can be time-consuming, subjective, and prone to errors^4^. In recent years, there has been a growing interest in leveraging the power of language models and deep learning techniques to develop automated systems capable of predicting diseases directly from symptom descriptions. These advanced models have the potential to revolutionize healthcare by enabling early disease detection, facilitating prompt medical attention, and providing remote diagnosis and treatment recommendations^5–8^.

One promising approach to tackle this challenge is to harness the capabilities of language models, such as BERT (Bidirectional Encoder Representations from Transformers), which have demonstrated remarkable success in various natural language processing tasks. Language models like BERT can learn contextual representations of words and sentences, capturing the intricate relationships between symptoms and diseases. By training these models on large medical text corpora, they can acquire domain-specific knowledge and improve disease prediction accuracy^9–11^.

To further enhance the predictive capabilities of the language model, a bidirectional LSTM (Long Short-Term Memory) layer can be incorporated. The bidirectional LSTM allows the model to capture both past and future context, enabling a better understanding of the symptom descriptions and their relevance to specific diseases. The bidirectional LSTM, coupled with the powerful representation learning of BERT, forms a robust framework for accurate disease prediction^12–14^.

However, developing an optimal language model architecture and determining the best hyperparameters can be a complex and time-consuming process. To address this challenge, the Hyperopt library can be employed. Hyperopt utilizes a Bayesian optimization algorithm^13^ (such as TPE—Tree-structured Parzen Estimator) to efficiently search through a predefined hyperparameter space and identify the optimal configuration for the language model. This automated hyperparameter tuning greatly enhances the model's performance and generalizability^15,16^. Accurate and timely diagnosis of diseases based on symptom descriptions is a critical aspect of effective healthcare delivery. However, traditional diagnostic approaches heavily rely on the expertise and experience of medical professionals, which can be subjective, time-consuming, and error prone. Furthermore, the increasing volume of medical literature and the complexity of disease manifestations pose significant challenges for accurate disease diagnosis. To address these challenges, there is a need for automated systems that can predict diseases directly from symptom descriptions, leveraging the power of language models and deep learning techniques. The objective is to develop a robust and accurate model that can assist healthcare professionals in making timely and precise diagnoses, leading to improved patient outcomes. The existing approaches to disease prediction from symptoms often suffer from limitations. Conventional machine learning algorithms can struggle to capture the intricate relationships and context between symptoms and diseases, leading to suboptimal predictive performance. Additionally, manually designing the architecture and selecting hyperparameters for these models can be a time-consuming and resource-intensive process. To overcome these limitations, our study focuses on developing an advanced language model for disease prediction, specifically utilizing the MCN-BERT + AdamP and MCN-BERT + AdamW architectures. These models combine the power of BERT's contextual embeddings, bidirectional LSTM layers, and hyperparameter optimization using Hyperopt to improve disease prediction accuracy.

Despite the growing interest in leveraging language models and deep learning techniques for disease prediction from symptom descriptions, there is a lack of comprehensive studies that integrate the power of BERT's contextual embeddings, bidirectional LSTM layers, and automated hyperparameter optimization using Hyperopt in the field of healthcare. Existing research often focuses on individual components or employs simpler models, without fully exploring the potential of these advanced techniques. Therefore, there is a research gap in developing a robust and accurate language model architecture specifically designed for disease diagnosis. The motivation behind this research is to address the limitations of traditional diagnostic methods in healthcare, which can be time-consuming, subjective, and prone to errors. By leveraging the power of language models and deep learning techniques, there is an opportunity to revolutionize disease diagnosis by enabling early detection, prompt medical attention, and remote diagnosis and treatment recommendations.

The proposed research aims to harness the capabilities of BERT's contextual embeddings, bidirectional LSTM layers, and hyperparameter optimization using Hyperopt to develop an accurate and efficient language model for disease prediction from symptom descriptions.

There are several challenges in developing an optimal language model architecture for disease diagnosis. Firstly, the selection and integration of suitable components such as BERT, bidirectional LSTM, and hyperparameter optimization algorithms require careful consideration to ensure compatibility and maximize performance. Secondly, training and fine-tuning large language models like BERT on medical text corpora can be computationally expensive and time-consuming. Handling such computational challenges efficiently is crucial. Additionally, the evaluation and validation of the proposed models need to be conducted rigorously, comparing them against existing methods and considering real-world healthcare scenarios. The main contributions of this research are as follows:

1. Develop an effective language model that can accurately predict diseases from symptom descriptions.
2. Investigate the performance of the MCN-BERT + AdamP and MCN-BERT + AdamW architectures in disease prediction using two distinct datasets.
3. Explore the impact of incorporating bidirectional LSTM layers to capture the contextual relationships between symptoms and diseases.
4. Apply hyperparameter optimization using Hyperopt to enhance the model's performance and generalize well to unseen data.

By addressing these goals, we aim to provide healthcare professionals with a reliable and efficient tool for disease diagnosis. This enables early detection, prompt intervention, and personalized treatment recommendations, ultimately leading to improved patient care and outcomes. In the following sections, we describe the methodology used to develop the language model, including data preprocessing, model architecture, and hyperparameter optimization. We present the experimental results, evaluate the performance of the proposed models, and compare them with existing approaches. The reminder of this paper is organized as follows. Section "Related work" states the current efforts and the related work for automating disease prediction from symptoms. Section "Preliminaries" investigtes the preliminaries, and Section "Proposed work" shows the proposed MCN-BERT method. The experimental results is demonstrated in Section "Experimental and results". The Discussion, limitation and conclusions are shown in sections "Discussion", "Limitations", and “Conclusion and future work”, respectively.

## Related work

The literature studies have explored various approaches and methodologies for detecting adverse drug reactions (ADRs) to ensure patient safety and optimize medication outcomes. In this section, the advent of deep learning models like BERT (Bidirectional Encoder Representations from Transformers) has significantly advanced this field in recent years. Molina et al.^17^ enhances DDI relationship extraction using two models with a Gaussian noise layer. The PW-CNN model captures pharmacological entity relationships in biomedical databases, while the BERT language model classifies and integrates data from target entities.

The experiment shows improved performance compared to previous models. Machado et al.^18^ highlight the use of Natural Language Processing (NLP) and machine learning classifier training to extract drug-drug interactions from unstructured data, supporting clinical prescribing decisions. The proposed system generates structured information from three data sources, identifying drug entities and determining interactions. Nguyen et al.^19^ investigate the Relation Bidirectional Encoder Representations from Transformers (Relation BERT) architecture for detecting and classifying DDIs in biomedical texts. Three models, R-BERT ∗ , RBioBERT1, and R-BioBERT2, achieve a macro-average F1-score of over 79% and 90.63% and 97% accuracy respectively, promoting the widespread application of automatic DDI extraction. KafiKang et al.^20^ presents a novel solution using Relation BioBERT (R-BioBERT) and Bidirectional Long Short-Term Memory (BLSTM) to detect and classify Drug-Drug Interactions (DDIs), enhancing prediction accuracy and identifying specific drug interaction types, with higher F-scores. Yang et al.^21^ proposes CAC model is a multi-layer feature fusion text classification model that combines CNN and attention. It extracts local features and calculates global attention, drawing inspiration from membrane computing. Experimental results show that the CAC model outperforms models relying solely on attention and exhibits significant improvements in accuracy and performance compared to other models.

Chaichulee et al.^22^ evaluated three NLP techniques—Naive Bayes-Support Vector Machine (NB-SVM), Universal Language Model Fine-tuning (ULMFiT), and various pre-trained BERT models including mBERT, XLM-RoBERTa, WanchanBERTa, and a domain-specific AllergyRoBERTa model trained on a dataset of 79,712 drug allergy records reviewed by pharmacists—to identify symptom terms from clinical notes, finding that while the BERT models generally demonstrated the highest performance, the NB-SVM model outperformed ULMFiT and BERT for less frequently coded symptoms. An ensemble model combining the different algorithms achieved strong results with 95.33% exact match ratio, 98.88% F1 score, and 97.07% mean average precision for the 36 most frequent symptoms, and this developed model was further enhanced into a symptom term suggestion system that tested well in prospective pharmacist trials with a 0.7081 Krippendorff's alpha agreement coefficient, indicating reasonably high agreement between the model's suggestions and pharmacist assessments.

Lee et al.^23^ proposed BioBERT, a specialized language representation model designed for biomedical text mining which is pre-trained on large-scale biomedical corpora. BioBERT was found to exhibit superior performance compared to BERT as well as previous state-of-the-art models on various biomedical text mining tasks, significantly surpassing baseline models in biomedical named entity recognition with a 0.62% F1 score improvement, biomedical relation extraction with a 2.80% F1 score improvement, and biomedical question answering with a 12.24% MRR improvement. In contrast, while BERT performed similarly to previous models, the analysis indicated that pre-training BERT on biomedical data enhances its ability to comprehend complex biomedical texts, demonstrating BioBERT's advantages for biomedical natural language understanding tasks over baselines.

The main objective of Huang et al.^24^ is to create and assess a continuous representation of clinical notes in order to predict 30-day hospital readmission at different stages of admission, including early stages and at discharge. They utilize bidirectional encoder representations from transformers (BERT) for analyzing clinical text. Since publicly available BERT parameters are trained on standard corpora like Wikipedia and BookCorpus, which differ from clinical text, they pre-train BERT using clinical notes and fine-tune the network specifically for predicting hospital readmission. This results in the development of ClinicalBERT. ClinicalBERT demonstrates superior performance compared to various baseline models in predicting 30-day hospital readmission, utilizing both discharge summaries and the initial days of notes in the intensive care unit, based on clinically relevant metrics. Additionally, the attention weights of ClinicalBERT can be utilized to interpret the predictions made by the model, providing valuable insights. Their model achieved Area Under the Receiver Operation Curve 0.714 based on the clinical BERT.

Hazell and Shakir^25^ conducted a review to assess the extent of under-reporting of adverse drug reactions (ADRs) in spontaneous reporting systems. A literature search identified 37 studies from 12 countries using diverse methodologies like hospitals and general practices, which provided 43 estimates of under-reporting calculated as the percentage of ADRs detected but not reported. The median under-reporting rate across studies was 94% with an interquartile range of 82–98%, with no significant difference in medians between general practice and hospital studies. However, general practice studies indicated a higher median under-reporting rate for all ADRs versus more serious/severe ADRs, while hospital studies consistently showed high medians for serious/severe ADRs. Studies of specific serious/severe ADR-drug combinations had a lower but still high median under-reporting rate of 85%.

Putra et al.^26^ presents a digestive system in processing daily consumed food and drinks. Their challenges are lack of awareness and knowledge about initial symptoms of digestive diseases can lead to serious complications, even death. Early identification of symptoms is essential for timely diagnosis and implementing control measures to prevent disease spread. The anamnesis process involves gathering disease symptoms through patient-medical personnel interactions, which are recorded in Electronic Medical Records (EMRs) to aid Clinical Decision Support (CDS). However, EMRs often pose challenges for computational processing due to grammar inconsistencies. To enable computers to process natural languages, Natural Language Processing (NLP) techniques are employed. This study focuses on developing an NLP system to identify symptoms of digestive diseases, optimizing the CDS process. Named Entity Recognition (NER) is utilized to determine tokens associated with disease symptoms. Through training the model with 50 epochs, an F1-score accuracy of 0.79 is achieved. Experimental results demonstrate that NER, supported by stemming and stopwords removal in pre-processing, enhances system accuracy. The summary of the current efforts of recent Advances in ADR detection using Machine Learning Algorithms is shown in Table 1.

**Table 1:** Summary of ADR detection studies using deep learning.

Author	Dataset used	Methodology	Results	Comment
Molina et al.^17^	Unstructured biomedical literature	Deep learning models PW-CNN + BERT	Improved performance compared to previous models	Proposed a novel framework for DDI relationship extraction using two deep learning models, PW-CNN and BERT
Machado et al.^18^	Electronic medical records	NLP and machine learning classifier training	Supports clinical prescribing decisions	Developed a system to generate structured information from three data sources, identifying drug entities and determining interactions
Nguyen et al.^19^	Biomedical texts	Relation Bidirectional Encoder Representations from Transformers (Relation BERT) architecture	Macro-average F1-score of over 79% and 90.63% and 97% accuracy	Proposed a novel architecture for detecting and classifying DDIs in biomedical texts
KafiKang et al.^20^	Unstructured biomedical texts	Relation BioBERT (R-BioBERT) and Bidirectional Long Short-Term Memory (BLSTM)	Enhanced prediction accuracy and identified specific drug interaction types	Presented a novel solution using R-BioBERT and BLSTM to detect and classify DDIs
Yang et al.^21^	Biomedical texts	CAC model: a multi-layer feature fusion text classification model that combines CNN and attention	Outperforms models relying solely on attention and exhibits significant improvements in accuracy and performance	Proposed a novel CAC model that combines CNN and attention to detect and classify DDIs
Chaichulee et al.^22^	79,712 drug allergy records	Naive Bayes—Support Vector Machine (NB-SVM), Universal Language Model Fine-tuning (ULMFiT), and Bidirectional Encoder Representations from Transformers (BERT)	Ensemble model achieved strong results, including an exact match ratio of 95.33%, an F1 score of 98.88%, and a mean average precision of 97.07%	Presented a dataset of drug allergy records and evaluated three NLP techniques for detecting drug allergies. BERT models demonstrated the highest performance
Lee et al.^23^	Biomedical corpora	BioBERT (Bidirectional Encoder Representations from Transformers for Biomedical Text Mining)	Exhibits superior performance compared to BERT and previous state-of-the-art models in various biomedical text mining tasks	Proposed a specialized language representation model, BioBERT, for biomedical applications
Huang et al.^24^	Clinical notes	Bidirectional encoder representations from transformers (BERT)	Superior performance compared to various baseline models in predicting 30-day hospital readmission	Developed ClinicalBERT, a pre-trained BERT model on clinical notes, for predicting 30-day hospital readmission
Hazell and Shakir^25^	Systematic literature search	NA	Median under-reporting rate of 94%, with an interquartile range of 82–98%	Reviewed the extent of under-reporting of ADRs in spontaneous reporting systems
Putra et al.^26^	Electronic medical records	Named Entity Recognition (NER)	f1-score accuracy of 0.79	Developed an NLP system to identify symptoms of digestive diseases, optimizing the CDS process

## Preliminaries

### BERT NLP optimization model

BERT is a widely used open-source natural language processing platform that stands for Bidirectional Encoder Representations from Transformers. Developed to help machines better comprehend ambiguous meanings in text or masked words in queries, BERT employs a transformer architecture where each output element attends to every input through learned weightings to capture relationships, establishing context critical for natural language understanding. At its core, BERT leverages bidirectional transformers that allow information to flow both forward and backward through the model, enabling it to learn the full context of language. By understanding word interdependencies through bidirectional context, BERT can more accurately derive meanings, even when portions of text are removed. This ability to comprehend language holistically based on the entire input rather than just preceding words gives BERT strong performance on tasks like question answering, setting a new standard in NLP and making it a popular foundation for many natural language applications^27–30^.

BERT learns language representations using an unsupervised pre-training strategy on a huge dataset, allowing the model to comprehend the context of an input sentence. To achieve good results, the model can be fine-tuned after pre-training on a task-specific supervised dataset. The fine-tuning stage includes two strategies: fine-tuning and feature based. Elmo employs a feature-based model, in which the model architecture is task-specific, with each task employing a distinct model and pre-trained language representations. BERT comprehends language by utilizing bidirectional layers of transformer encoders, hence the name BERT. Unlike previous language models that generate context-free word embeddings, such as Glove2Vec and Word2Vec, BERT provides context by assessing the term's relationship with the terms that come before and after it^31,32^.

#### Bi-directional (B)

Prior to BERT, the models could only move the context window in one way. To grasp the context, it can either relocate the word to the left or right. BERT, on the other hand, employs bidirectional language modeling. According to contextual language modeling, BERT can see the entire sentence and move it right or left^33^.

#### Encoder representations (ER)

Any text that is passed via a language model can be encoded before being provided as input. Only the encoded text can be processed and yield a result. Any model's output can also be in encrypted format, which must be decrypted. As a result, once a message has been encoded, it must be decoded again. It is a two-way mechanism.

#### Transformers (T)

For text processing, BERT employs transformers and masked language modeling. The main challenge is comprehending the context of the word mentioned in that location. The pronouns in a phrase can be difficult for the machine to understand. Transformers can therefore pay attention to pronouns, try the word with the entire sentence, and comprehend the context. Masked language modeling prevents the target word from comprehending it. The mask prevents the word from diverging from its intended meaning. BERT can predict the missing word if the masking is in place, which is doable with fine-tuning. BERT operates by following the steps outlined below:

##### Step 1: Large amounts of training data

BERT is designed to process significantly longer word counts thanks to being trained on vast underlying data repositories, imbuing it with broad linguistic knowledge of English and other languages. While this capability enables BERT's powerful natural language understanding, it also means training the model on even larger datasets requires more computational resources and time due to BERT's transformer architecture. However, the model's training process can be accelerated using Tensor Processing Units, allowing BERT to leverage massive datasets during pre-training and fine-tuning to further enhance its abilities—demonstrating how its transformer design enables effective training even on big data, despite the demanding resources and time needed to optimize BERT's immense knowledge base derived from its enormous linguistic foundation.

##### Step 2: Masked language model

**Figure 1:**
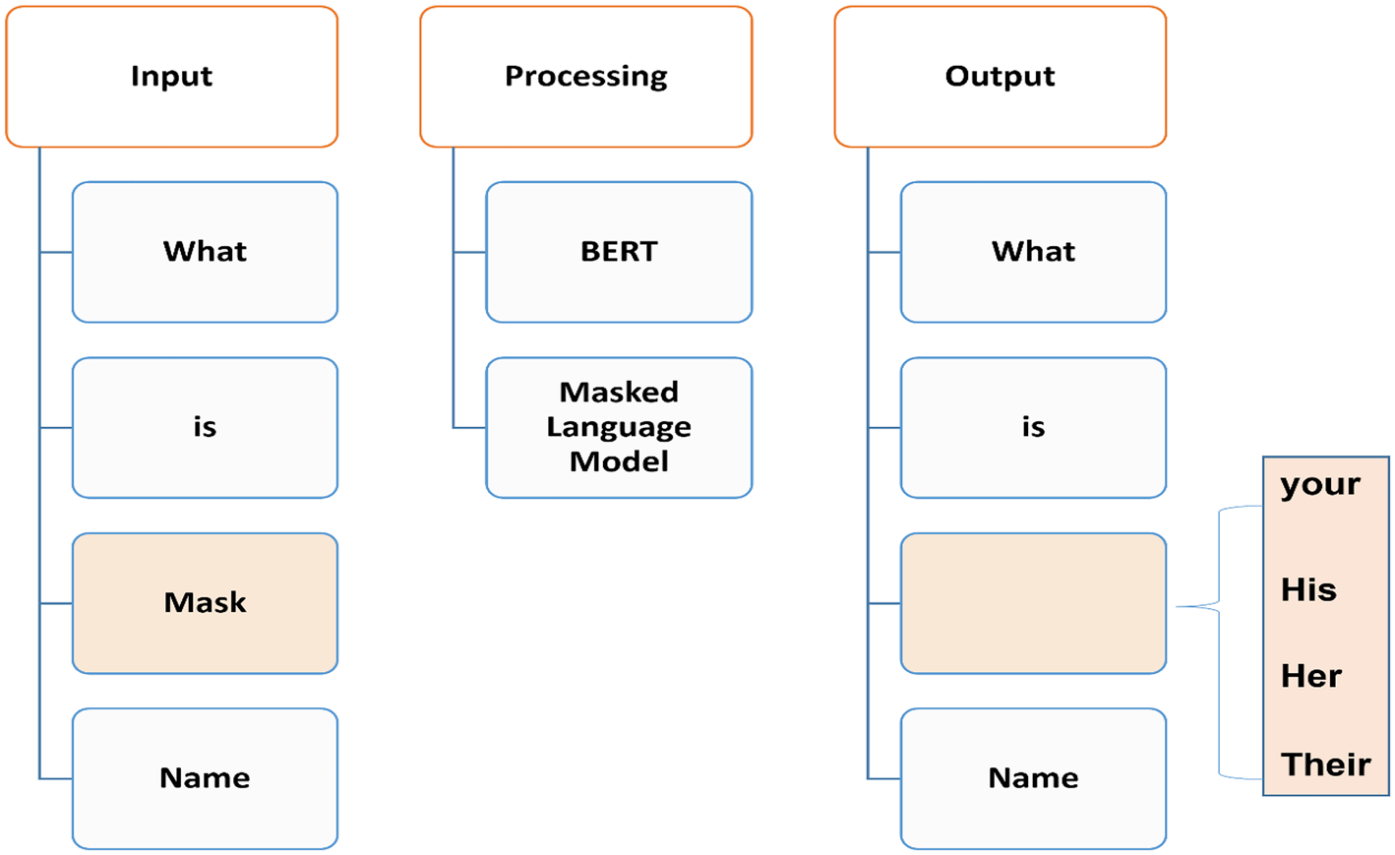
The Input Process Output (IPO) for the MLM model.

The Masked Language Model (MLM) objective enables BERT's ability to learn from text bidirectionally. This is accomplished through masking a word randomly in a sentence and requiring BERT to predict the masked word based on both preceding and following context words simultaneously. As illustrated in Fig. 1, by corrupting the input and challenging BERT to replace the masked word using its understanding of relationships between all other words in the sentence, the MLM approach allows information to flow in both directions—from left to right and right to left. This allows BERT to developed richer, contextually aware representations of language by comprehending the full semantic meaning derived from words surrounding the masked token, empowering it with a more comprehensive understanding of word usage and intended meaning within a passage of text.

By considering the word bidirectionally after and before the hidden text, we can readily predict the missing word. The bidirectional strategy utilized here can aid in achieving the best level of accuracy. During training, a random 15% of the tokenized words are masked, and BERT's duty is to guess the word.

##### Step 3: Next sentence prediction

**Figure 2:**
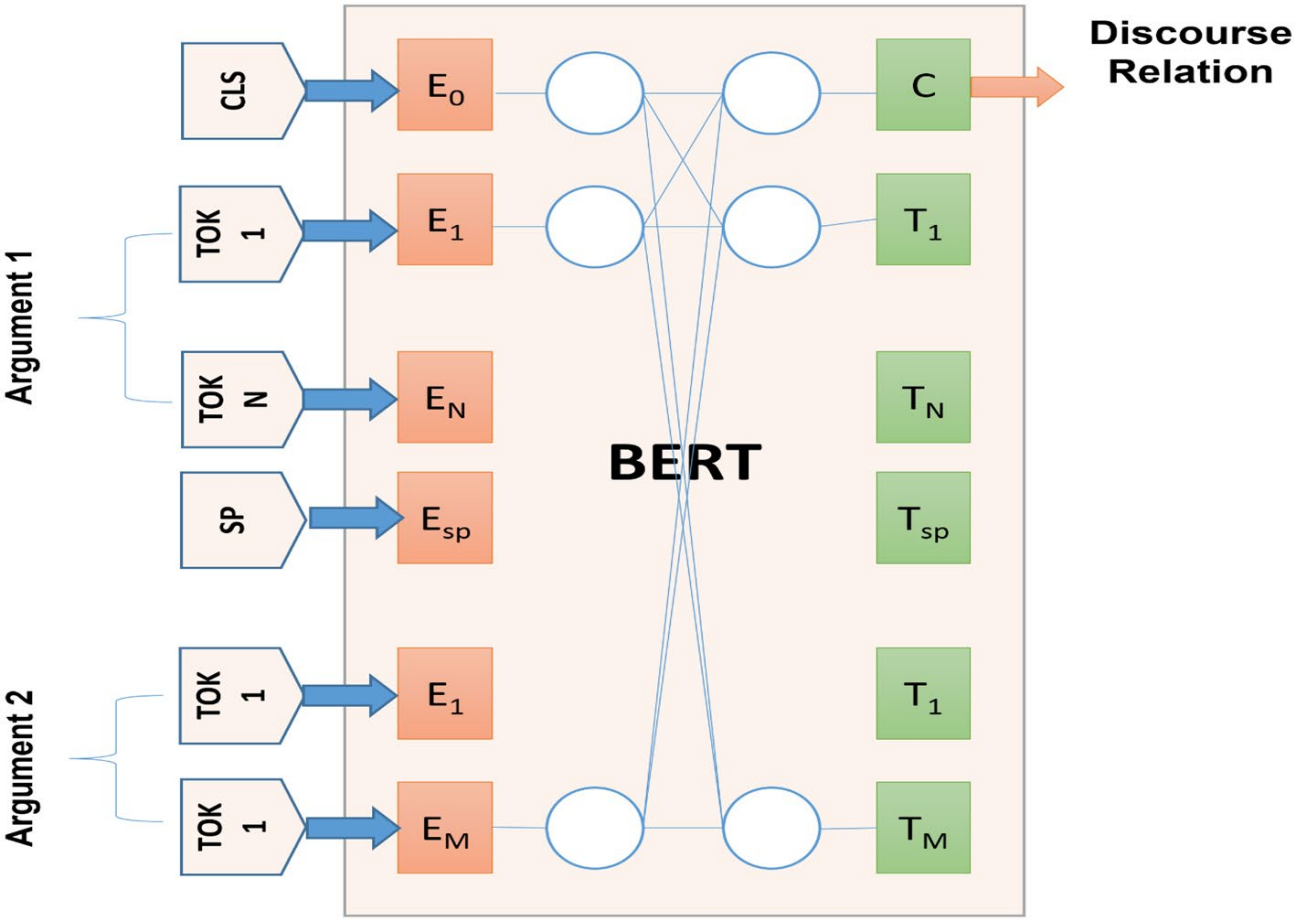
The BERT mechanism for two arguments and the resulting discourse relation.

Next Sentence Prediction (NSP) assists BERT in learning about sentence relationships by predicting whether a particular sentence follow the previous one. In training, 50% of successful predictions are fixed with 50% random words to assist BERT improve its accuracy, as illustrated in Fig. 2.

##### Step 4: Transformers

The transformer design efficiently parallelizes machine learning training. Massive parallelization enables the model to train BERT on large amounts of data quickly. Transformers operate by exploiting attention. It first appears in computer vision models and is a powerful deep-learning approach.

Because human brains have limited memory capacity, machine learning models must learn to focus on what is most important. When the machine learning model achieves this, we can avoid wasting computational resources and instead use them to process irrelevant information. Transformers generate differential weights by transmitting signals to the words in a sentence that are important for subsequent processing.

A transformer can accomplish this by correctly processing an input through transformer stack levels known as encoders. Another transformer layer stack called a decoder aid in output prediction. Transformers are well-suited for unsupervised learning because they can efficiently analyze more data points.

##### Step 5: Fine-tuning BERT

**Figure 3:**
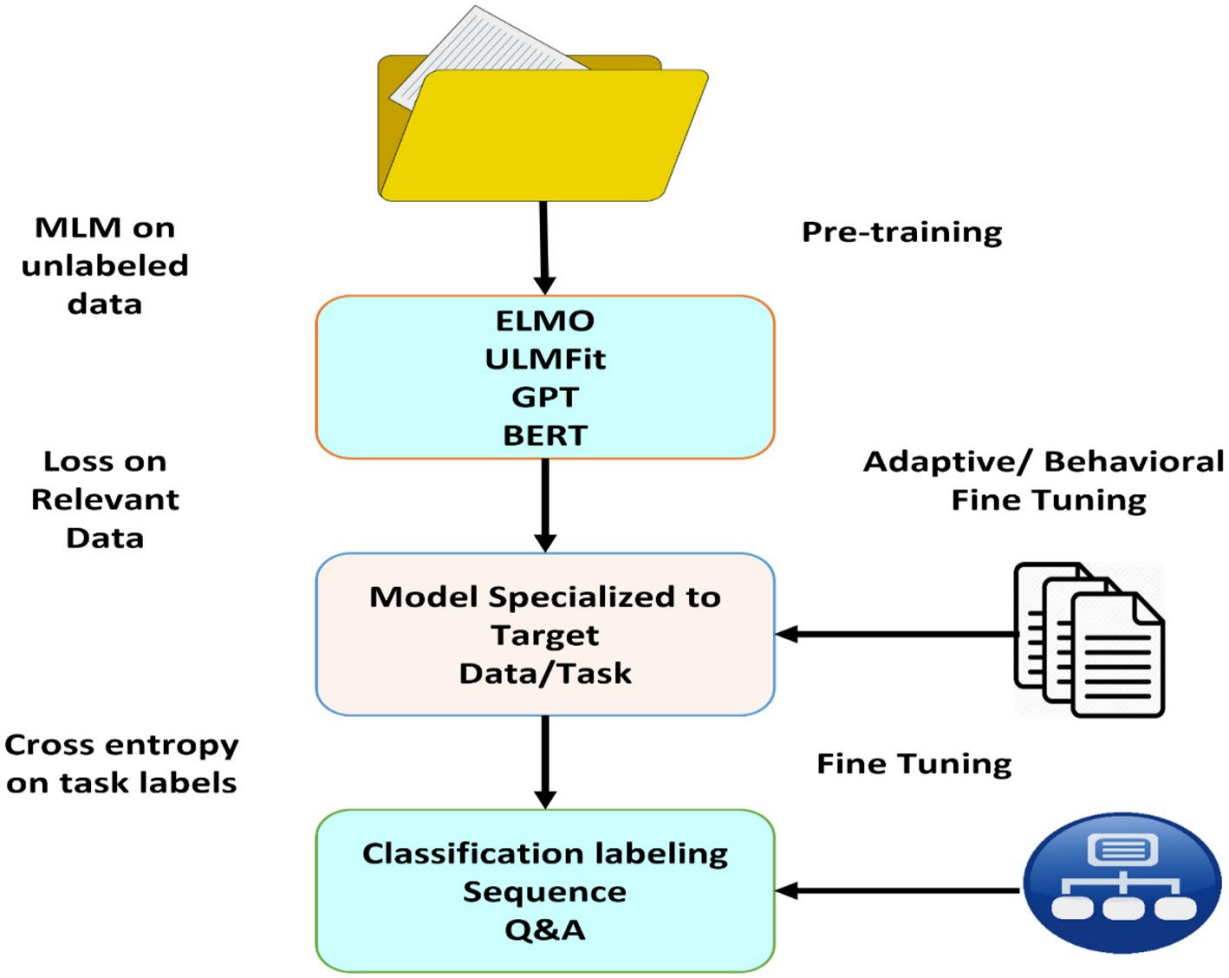
The steps of classification and sequence labeling BERT NLP.

The BERT NLP optimization model for text classification can be refined by first obtaining the dataset and exploring it using Pandas, examining word counts, labels, lengths, and densities. The dataset is then preprocessed on the CPU by preparing training data, tokenizing ids, obtaining the tokenizer and BERT layer, and preprocessing text for BERT. An input pipeline is created by transforming the train and test datasets. A BERT classification model is then developed, trained while monitoring on a few sets, and evaluated through supervised trials with various training graphs, metrics, and timings. The model can be updated, optimized, and saved using different technologies to ensure repeatability and performance improvements. Using these steps, we can fine-tune the BERT NLP optimization model for text classification as shown in Fig. 3.

### Advantages of the BERT language model

The BERT Language Model has several advantages over other models in terms of its architecture and pre-training:

- *Bidirectional training* BERT uses a bidirectional Transformer, allowing it to learn the context of words from both left and right. This gives BERT a better understanding of language.
- *Pre-trained on large corpus* BERT is pre-trained on the enormous Google Book corpus and Wikipedia using masked language modeling and next sentence prediction tasks. This massive pre-training gives BERT strong language understanding abilities.
- *Can be fine-tuned for downstream tasks* While other models require training from scratch for new tasks, BERT provides a general-purpose language representation that can be efficiently fine-tuned using just one additional output layer for specific NLP problems. This makes it easy to apply BERT to new tasks with limited data.
- *Multilingual support* In addition to English, BERT is also available pre-trained in over 100 languages, allowing it to be easily applied to projects in languages other than English with no additional training required.
- *State-of-the-art performance* BERT has achieved new performance highs on many NLP tasks, demonstrating its effectiveness at understanding relationships between words and contexts in language. It continues to be improved through updates by the authors to stay at the forefront of language modeling techniques.

### Disadvantages of the BERT language model

While BERT's massive pre-training provides it with strong language understanding abilities, its large size also presents some drawbacks:

- *High computational resources required* Training in the original BERTBASE model required 4 days using 16 Google TPUv3 chips. Fine-tuning BERT for new downstream tasks and larger models also requires significant computing power.
- *Slow for training* With billions of parameters, fine-tuning BERT is a very computationally intensive process that can take hours or days depending on the size of model and data. This slow training speed limits experimentation.
- *High memory usage* The BERT models, especially larger ones, require large amounts of memory/VRAM to handle their parameters during training and inference. This restricts their use of devices with limited memory.
- *Not optimized for inference* As an encoder designed for language understanding, BERT performs slower inferences compared to smaller task-specific models. Its efficiency for production use is limited compared to optimized classifiers.
- *Limits batch size* To fit in memory, smaller batch sizes must be used during training BERT compared to smaller models, making training less stable and slower to converge.

While pre-training provides advantages, the enormous size of BERT results in computational constraints that restrict its application depending on available hardware resources. Ongoing work aims to reduce this overhead through model compression.

### MCN-BERT + AdamP

The MCN-BERT model is a variant of the BERT model that uses a multi-layer self-attention mechanism to model the relationships between different parts of a sequence. The AdamP optimizer is a popular stochastic gradient descent algorithm that adapts the learning rate for each parameter based on the magnitude of the gradient.

#### Advantages

- *Improved performance* The MCN-BERT model has been shown to achieve state-of-the-art results on several natural language processing tasks.
- *Adaptive learning rate* The AdamP optimizer adapts the learning rate for each parameter based on the magnitude of the gradient, which can help to converge faster and avoid getting stuck in local minima.

#### Disadvantages

- *Computationally expensive* The MCN-BERT model is computationally expensive to train and use, which can be a challenge for applications with limited resources.
- *Requires pre-training* The MCN-BERT model requires pre-training on a large dataset of text, which can be time-consuming and can not be available for all languages or domains.

The loss function for the MCN-BERT + AdamP model can be written as in Eq. (1).

1 $$L = -\sum \left({y}_{true} *{\text{log}}\left({y}_{pred}\right)\right)$$

where y_true is the true label, y_pred is the predicted label, and the sum is taken over all examples in the dataset.

The optimization process for the MCN-BERT + AdamP model can be written as in Eq. (2).

2 $$AdamP\, (parameters) = media \,(0.9, 0.999, 0.001) * Adam\,(parameters)$$

where media is a function that calculates the mean of the weights and the standard deviation of the gradients, and Adam is a popular stochastic gradient descent algorithm that adapts the learning rate for each parameter based on the magnitude of the gradient.

### MCN-BERT + AdamW

The MCN-BERT + AdamW model is like the MCN-BERT + AdamP model but uses a different optimizer. The AdamW optimizer is a variant of the Adam optimizer that uses a different formula for calculating the learning rate for each parameter.

#### Advantages

- *Improved performance* The MCN-BERT + AdamW model has been shown to achieve state-of-the-art results on several natural language processing tasks.
- *Adaptive learning rate* The AdamW optimizer adapts the learning rate for each parameter based on the magnitude of the gradient, which can help to converge faster and avoid getting stuck in local minima.

#### Disadvantages

- *Computationally expensive* The MCN-BERT + AdamW model is computationally expensive to train and use, which can be a challenge for applications with limited resources.
- *Requires pre-training* The MCN-BERT + AdamW model requires pre-training on a large dataset of text, which can be time-consuming and cannot be available for all languages or domains.

### Bidirectional LSTM + Hyperopt

The Bidirectional LSTM model is a type of recurrent neural network that uses two LSTM layers to model the relationships between different parts of a sequence. The Hyperopt optimizer is a meta-optimizer that uses a combination of different optimization techniques to optimize the model's parameters.

#### Advantages

- *Improved performance* The Bidirectional LSTM model has been shown to achieve state-of-the-art results on several natural language processing tasks.
- *Flexible* The Hyperopt optimizer can be used to optimize a wide range of models and hyperparameters, which makes it a flexible choice for a variety of applications.

#### Disadvantages

- *Computationally expensive* The Bidirectional LSTM model is computationally expensive to train and use, which can be a challenge for applications with limited resources.
- *Requires careful tuning* The Hyperopt optimizer requires careful tuning of the hyperparameters, which can be time-consuming and may not be available for all applications.

### Dataset description

In this section, we rigorously evaluate the efficacy of our proposed methodology by conducting experiments on two distinct datasets with varying structures. This validation process aims to demonstrate the resilience and versatility of our approach in diverse real-world scenarios. The two datasets described are distinct in terms of their composition, characteristics, and objectives. The first dataset is a collection of 1200 data points, each representing a unique combination of a disease label and a natural language symptom description. It has two columns, "label" and "text", with the "label" column containing disease labels and the "text" column containing natural language symptom descriptions. The dataset covers 24 distinct diseases, with 50 symptom descriptions for each disease, resulting in a total of 1200 data points. While, the second dataset aims to develop cutting-edge methods for automatically identifying Adverse Drug Reactions (ADRs) from Twitter data. To achieve this, a dataset consisting of 23,516 rows can be created, where each row represents a tweet that has been categorized as either ADR (1) or Non-ADR (0), based on the presence of drug names, symptoms, and effects. This dataset can enable Company X to monitor ADRs efficiently and accurately in real-time, allowing them to respond promptly to emerging health concerns and protect public health. Therefore, while the first dataset focuses on disease labels and symptom descriptions, the second dataset focuses on ADRs and their related tweets.

#### Dataset-1: Symptom2disease

The dataset consists of 1200 datapoints and has two columns: (i) label: contains the disease labels. (ii) text: contains the natural language symptom descriptions. The dataset comprises 24 different diseases, and each disease has 50 symptom descriptions, resulting in a total of 1200 datapoints. Table 2 illustrates the main content for the different diseases that have been covered in the dataset.

**Table 2:** The features indicates the number of diseases in Dataset-1.

Diseases	Description
Psoriasis	I have been experiencing a skin rash on my arms, legs, and torso for the past few weeks. It is red, itchy, and covered in dry, scaly patches
Varicose	As I am overweight, I have noticed that my legs are swollen, and the blood vessels are more visible than usual. The swelling seems to be getting worse over time
Typhoid	Because of the vomiting and diarrhea, I've been having a lot of difficulties staying hydrated. There is a mild fever, too, as well as stomach pain
Chicken pox	Enlarged lymph nodes are giving me a great deal of pain. I have rashes all over my body and because of which I cannot sleep all night
drug reaction	I no longer want to have sex, and it's difficult for me to do so. I regularly have brain fog and a sense of confusion

Dataset-1 was sourced from Kaggle, specifically from the following URL: https://www.kaggle.com/datasets/niyarrbarman/symptom2disease/. This dataset focuses on the relationship between symptoms and diseases. It comprises a collection of symptom-disease pairs, where each pair indicates the presence of a symptom and the corresponding disease. The dataset was curated by Niyarr Barman and made available on Kaggle. Regarding data curation processes, the dataset was compiled by extracting information from various reliable medical sources, including research papers, medical literature, and clinical databases. The process involved careful extraction and validation of symptom-disease associations to ensure data accuracy.

#### Dataset-2: Twitter Drug

The objective is to create innovative automated techniques for identifying Adverse Drug Reactions (ADRs) by analyzing social media data from Twitter. This endeavor seeks to address a crucial need in healthcare by mitigating the potential harm to patient health and alleviating the strain on healthcare systems that can automatically segment tweets into two categories: ADR(1) and NON-ADR(0) with 23,516 rows, based on mentions of the drug, associated symptoms, and observed effects. This segmentation can enable Company X to efficiently monitor and assess potential ADRs in real-time, enhancing their ability to respond to emerging health concerns effectively as shown in Table 3.

**Table 3:** The description of Dataset-2 features.

ID	Tweets	Label
413,205	Intravenous azithromycin-induced ototoxicity	1
528,244	Immobilization, while Paget's bone disease was present, and perhaps enhanced activation of dihydrotachysterol by rifampicin, could have led to increased calcium-release into the circulation	1
361,834	Unaccountable severe hypercalcemia in a patient treated for hypoparathyroidism with dihydrotachysterol	1
994,547	In all cases, ACE-inhibitor therapy either predisposed the patient to or precipitated the acute event	0

The tweets were collected from the public Twitter API using keywords related to common drugs and their known adverse effects. A panel of 3 clinical pharmacists manually categorized each tweet as indicating an adverse drug reaction (ADR = 1) or not (NON-ADR = 0).

Only tweets written in English containing drug names from the top 500 prescribed medications in the US were included. Retweets and non-original content were excluded. Inter-annotator agreement for the categorization task was calculated using Fleiss' kappa and found to be 0.82, indicating good reliability between raters.

Any tweets with discrepant labels were adjudicated through group discussion until consensus was reached. Of the total 23,516 tweets collected, 8289 (35.2%) were labeled as ADR and 15,227 (64.8%) as NON-ADR.

We hope this additional context provides needed transparency regarding the dataset construction process. Please let me know if any part of the annotation methodology requires further elaboration. Thank you for taking the time to ensure strong methodological reporting—it will certainly help improve our work.

Dataset-2 Original Link: https://www.kaggle.com/datasets/pawan2905/tweet-classification?select=Data.csv

Regarding validation for reliability and relevance, both datasets underwent rigorous validation processes. For Dataset-1, the symptom-disease associations were cross-checked with existing medical knowledge and validated by domain experts. For Dataset-2, the methods for detecting ADRs on Twitter were validated using benchmark datasets and established evaluation metrics. These validation steps were crucial to ensuring the reliability and relevance of the datasets in the context of disease prediction and ADR detection.

## Proposed work

**Figure 4:**
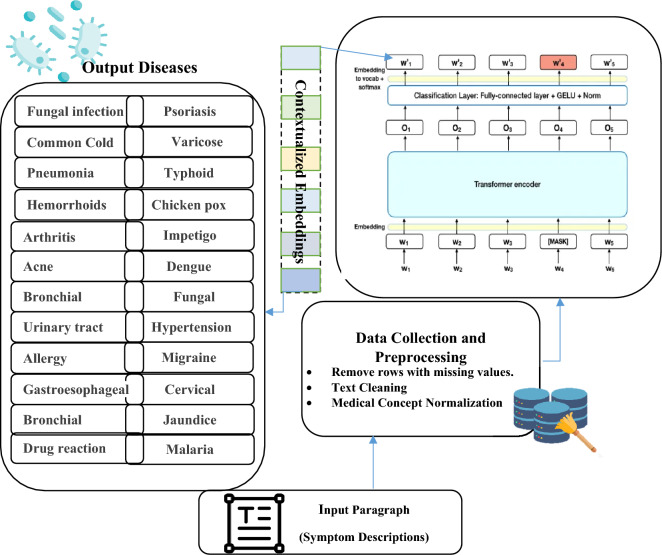
MCN-BERT proposed work architecture.

In this section, we propose a robust architecture called the Medical Concept Normalization—Bidirectional Encoder Representations from Transformers (MCN-BERT) model and BiLSTM model that illustrated in Algorithm 1. BERT, a deep contextual language model, effectively comprehends text context and semantics. When combined with medical concept normalization, it accurately maps medical terms to standardized concepts. MCN reduces ambiguity in medical terminology by mapping diverse expressions to a standardized code, improving precision in text classification tasks like disease diagnosis and drug recognition. BERT and Medical Concept Normalization automate the manual normalization of medical concepts, saving healthcare professionals time and reducing errors. The MCN-BERT model consists of the main components as follows: (i) Data collection and processing (ii) BERT Model and Tokenizer Initialization (iii) Model training. (iv) Model evaluation. (v) BiLSTM model architecture. Figure 4 and Algorithm 1 show the main steps for our MCN-BERT model.

**Figure:**
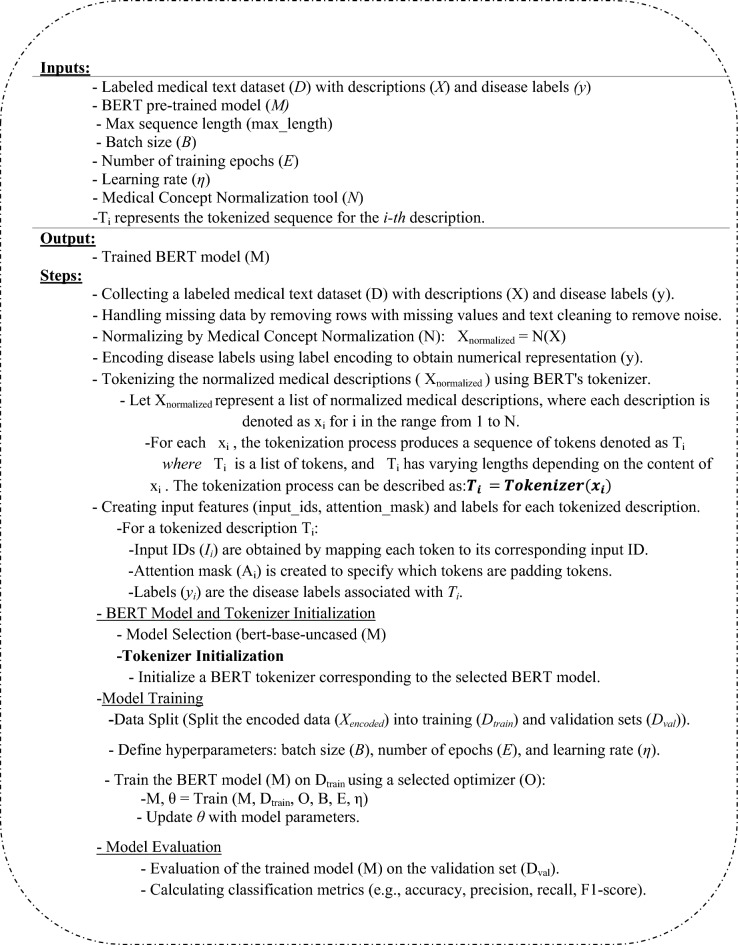
**Algorithm 1** The MCN-BERT proposed work main steps.

### Data collection and processing

In the initial stages of the MCN-BERT proposed work architecture, focus on handling the input datasets, which encompassed detailed symptom descriptions paired with corresponding disease labels. A preprocessing step involved the meticulous tokenization of these symptom descriptions, achieved through the utilization of a specialized medical tokenizer designed for enhanced contextual understanding of medical terms. We assigned numerical labels to diseases, a fundamental step for effective model training.

### BERT model and tokenizer initialization

Incorporating pre-trained BERT model weights is a foundational step in our model architecture. These pre-trained weights encapsulate extensive linguistic knowledge, enhancing the model's ability to comprehend intricate medical language. The architecture of the BERT model involves multiple transformer encoder layers to facilitate the effective processing of symptom descriptions, a specialized medical tokenizer is initialized. The tokenizer is designed to handle the nuances of medical terminology, ensuring accurate representation during tokenization. Consequently, the symptom descriptions are seamlessly converted into tokens, ready for integration into the BERT model as input, marking a pivotal stage in the model architecture.

### Model training

The training pipeline involves meticulous steps to ensure the effective learning of the MCN-BERT model. In the initial phase of Training Data Preparation, batches of tokenized symptom descriptions and corresponding disease labels are organized is represented as in Eq. (3).

3 $$Batch Data=PrepareBatches(TokenizedSymptoms,DiseaseLabels)$$

*where* $Batch Data$ signifies the prepared training batches, and $PrepareBatches$ denotes the function orchestrating this preparation. Subsequently, the Model Forward Pass is executed by conducting a forward pass through the BERT model for each batch. The contextualized embeddings for symptom descriptions are obtained, denoted as in Eq. (4).

4 $$Embeddings=BERT\_Model(Batch\_Data)$$

To normalize medical concepts in the embeddings, the Medical Concept Normalization (MCN) Layer is applied, ensuring consistency across different expressions of the same medical concept. Mathematically, this normalization is expressed as in Eq. (5).

5 $$Normalized\_Embeddings=MCN\_Layer(Embeddings)$$

The Prediction Head is then introduced to the BERT model to output disease predictions, incorporating additional trainable parameters specific to the disease prediction task. The subsequent Loss Calculation involves determining the loss between predicted disease probabilities and actual labels, defined as in Eq. (6).

6 $$L=LossFunction(PredictedProbabilities,ActualLabels)$$

*where* $L$ represents the calculated loss. This loss function, such as categorical cross-entropy, ensures accurate optimization during training. The final steps encompass Backpropagation and Optimization, where backpropagation computes gradients of the loss with respect to model parameters. The proposed model parameters are updated using an (AdamW, AdamP) optimization algorithms to minimize the loss. This process is iterated over multiple batches for a specified number of epochs, constituting the Training Iterations and ultimately leading to the trained MCN-BERT model.

### Model evaluation

The quality of the models was gauged based on well-known evaluation metrics such as the accuracy of the classification, precision, recall, and F1-scores for classification.

Equations (7), (8), (9), and (10) are determined the confusion matrix performance that represents the accuracy, precision, recall, F1-score, respectively^34–36^.

7 $$Accuracy=\frac{TP + TN}{TP + FP + TN + FN}$$

8 $$Precision=\frac{TP }{TP + FP}$$

9 $$Recall=\frac{TP }{TP + FN}$$

10 $$F1 - score =2* \frac{\left(Precision \times Recall\right)}{\left(Precision + Recall\right)}$$

These metrics are based on a “confusion matrix” that includes true positives (TP), true negatives (TN), false positives (FP), and false negatives (FN)^37^.

### BiLSTM model architecture

The Bidirectional Long Short-Term Memory (BiLSTM) models involves meticulous attention to data collection and processing, BiLSTM model architecture and tokenizer initialization, and the subsequent model training phase. In the initial phase of data handling, a diverse and well-labeled dataset is acquired, followed by rigorous preprocessing to address potential challenges such as missing values or inconsistencies. Symptom descriptions are tokenized and subjected to necessary transformations to ensure data quality and relevance. The BiLSTM model is then defined, specifying its architecture, including input layers, BiLSTM layers, and output layers. Crucially, the tokenizer is initialized to facilitate the conversion of textual data into a format suitable for ingestion by the BiLSTM model, involving the segmentation of text into individual tokens. Then, the model training process unfolds, beginning with the preparation of batches comprising tokenized symptom descriptions and corresponding disease labels. The selection of an appropriate loss function, such as categorical cross-entropy for multi-class classification tasks, is paramount. Additionally, optimizers and learning rates are chosen to govern the weight update mechanism during training. The actual training phase involves iteratively feeding batches into the BiLSTM model, enabling it to learn the mapping from symptom descriptions to disease labels. Continuous monitoring of training metrics, including loss and accuracy, aids in assessing the model's performance on both training and validation sets. Hyperparameter tuning, encompassing adjustments to parameters like epochs, batch size, and LSTM layer configurations, refines the model's effectiveness in predicting diseases from symptom descriptions. This comprehensive and iterative process ensures a methodical and optimized application of BiLSTM for disease prediction tasks.

## Experimental and results

To evaluate the effectiveness of our machine learning framework, we conducted experiments in this section. The experiments were performed on a computer with a 3 GHz i5 processor, 8 GB main memory, and 64-bit Windows 10 operating system. We used the Python programming language to carry out the experiment.

### The results of the proposed classification techniques

**Figure 5:**
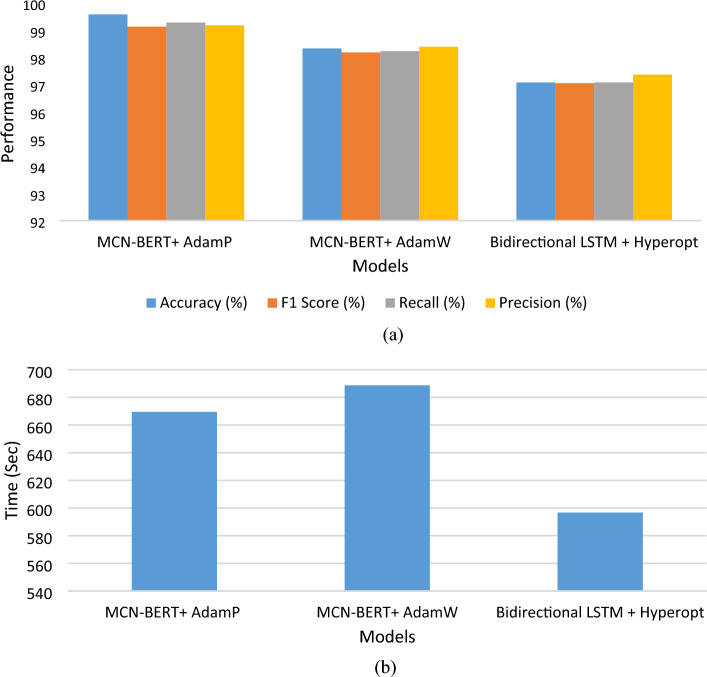
The performance of the proposed model for Dataset-1, (**a**) The evaluation metrics, (**b**) The total training time in Seconds.

**Figure 6:**
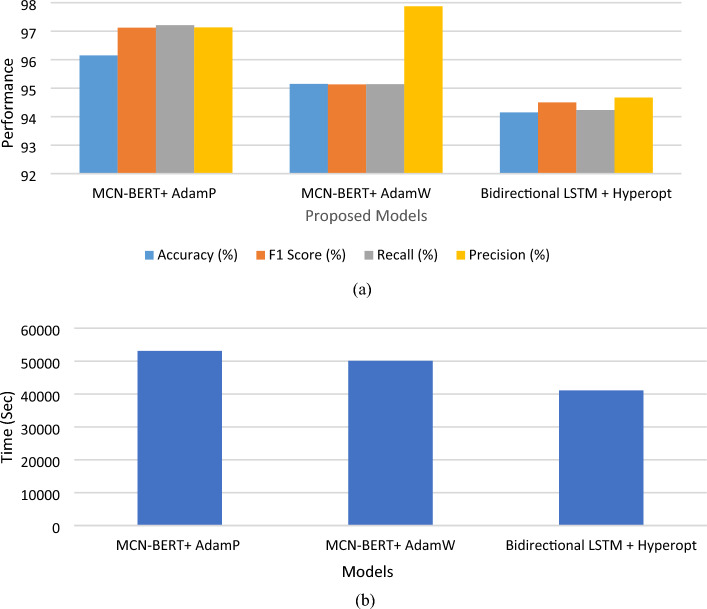
The performance of the proposed model for Dataset-2, (**a**) The evaluation metrics, (**b**) The total training time in Seconds.

Tables 4, and 5, and Figs. 5, and 6 represent the evaluation metrics for three different models: MCN-BERT + AdamP, MCN-BERT + AdamW, BiLSTM + Hyperopt, and the total training time in seconds for both Dataset-1 and Dataset-2, respectively. A comparative analysis of the proposed model and existing studies using Dataset-2 are shown in Table 6. The metrics include Accuracy, F1 Score, Recall, Precision, and Total Training Time. The analysis and expansion of the table can be represented as follows:

- *Model* This column shows the names of the machine learning models used in the classification task.
- *ROC AUC Score* This column represents the Receiver Operating Characteristic (ROC) Area Under the Curve (AUC) score, which measures the ability of the model to distinguish between positive and negative classes. A higher ROC AUC score indicates better performance.
- *Accuracy* This column represents the proportion of correctly classified samples. A higher accuracy indicates better performance.
- *Precision* This column represents the proportion of true positive samples among all positive samples. A higher precision indicates fewer false positives.
- *Recall* This column represents the proportion of true positive samples among all actual positive samples. A higher recall indicates fewer false negatives.
- *F1-score* This column represents the harmonic mean of precision and recall. A higher F1-score indicates a better balance between precision and recall.
- *Time Taken* This column represents the amount of time taken by each model to complete the classification task. The proposed MCN-BERT performance is shown in Fig. 5.

**Table 4:** The performance metrics of the proposed Models using Dataset-1.

Evaluation Metrics Dataset-1					Total Training Time (sec)
Model	Accuracy (%)	F1 Score (%)	Recall (%)	Precision (%)	
MCN-BERT + AdamP	99.58	99.13	99.28	99.18	669.50
MCN-BERT + AdamW	98.33	98.18	98.23	98.39	688.69
BiLSTM + Hyperopt	97.08	97.05	97.08	97.37	596.70

**Table 5:** The performance metrics of the proposed Models using Dataset-2.

Evaluation Metrics Dataset-2					Total training time (s)
Model	Accuracy (%)	F1 Score (%)	Recall (%)	Precision (%)	
MCN-BERT + AdamP	96.15	97.12	97.21	97.13	53,094.27
MCN-BERT + AdamW	95.15	95.13	95.14	97.87	50,094.34
BiLSTM + Hyperopt	94.15	94.50	94.23	94.67	41,094.15

**Table 6:** The Comparative study between the proposed model and some current studies.

Model	Accuracy (%)	F1 Score (%)	Recall (%)	Precision (%)
MCN-BERT + AdamP	96.15	97.12	97.21	97.13
MCN-BERT + AdamW	95.15	95.13	95.14	97.87
Bidirectional LSTM + Hyperopt	94.15	94.50	94.23	94.67
Nguyen et al.^17^	97.00	90.63	N/A	N/A
Chaichulee et al.^20^	95.33	98.88	N/A	97.07
Hazell et al.^23^	94.00	98.00	N/A	N/A

### Model training

**Figure 7:**
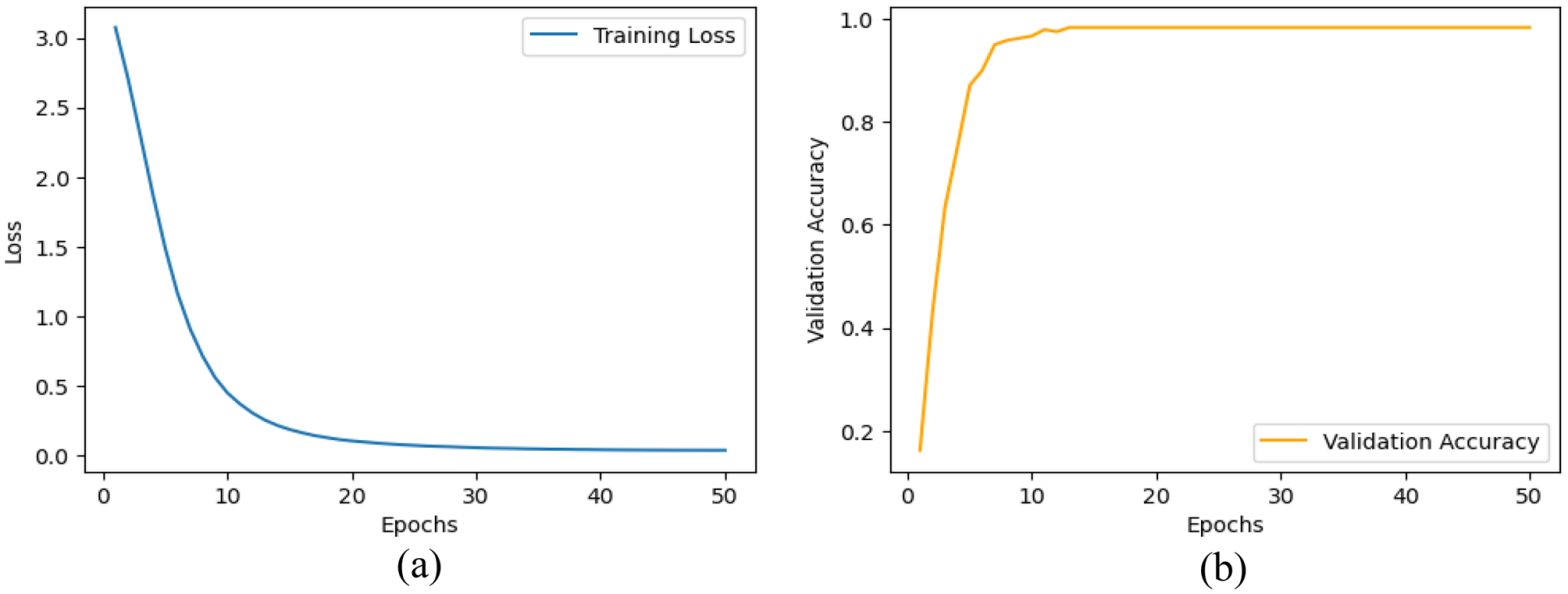
The performance of the proposed method with AdamP optimizer (**a**) The training loss, and (**b**) The validation accuracy for Dataset-1.

**Figure 8:**
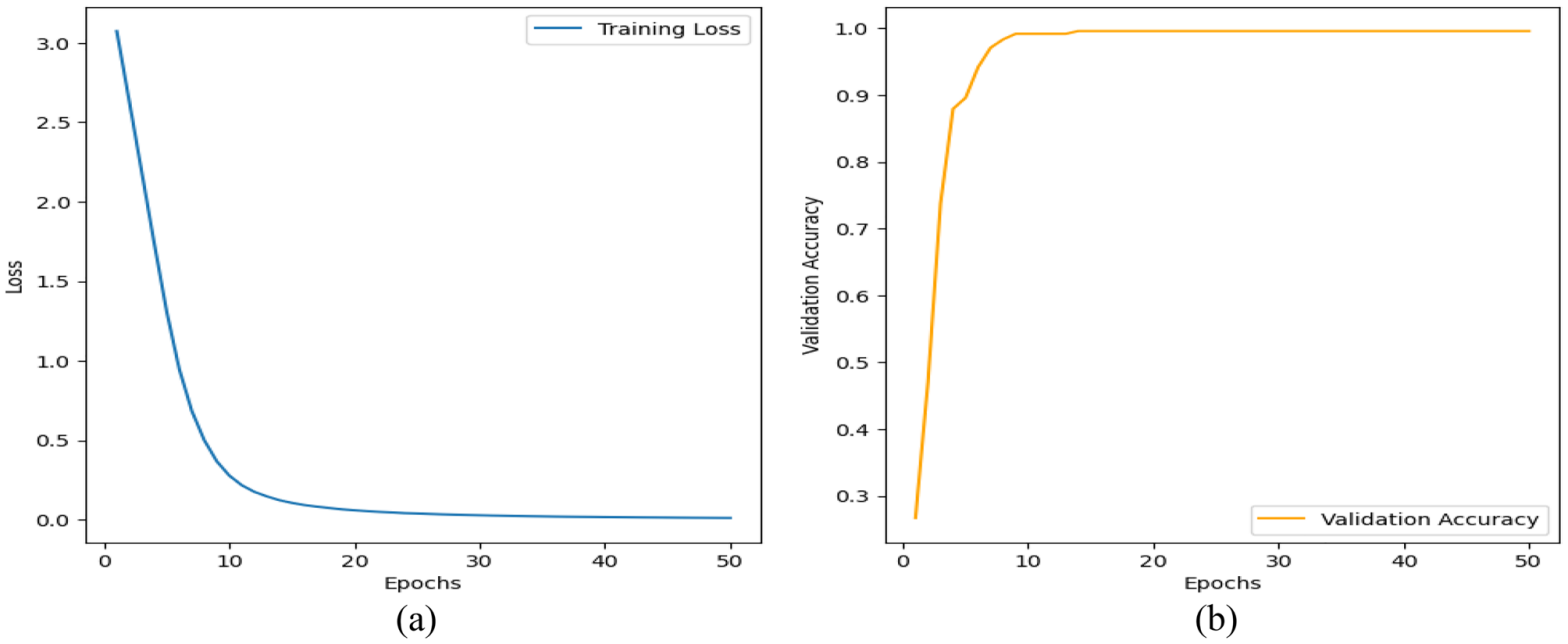
The performance of the proposed method with AdamW optimizer. (**a**) The training loss, and (**b**) The validation accuracy for Dataset-1.

**Figure 9:**
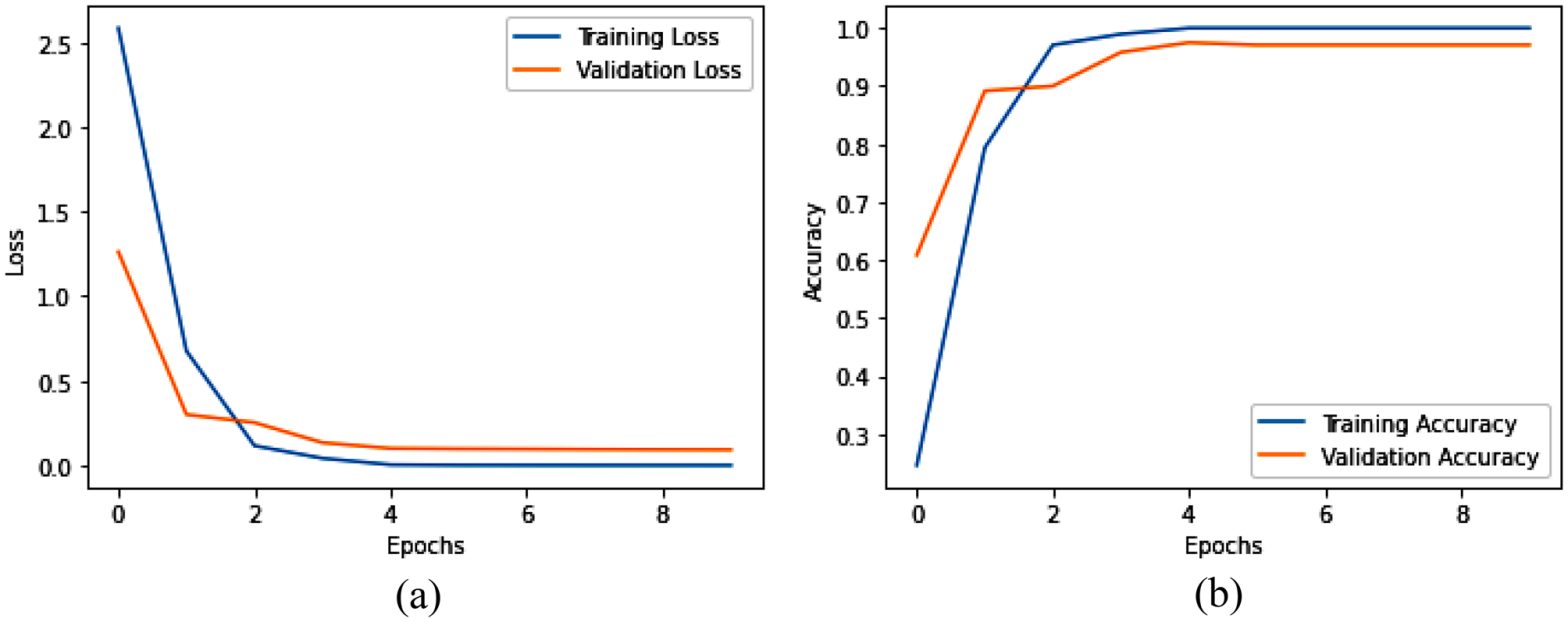
The performance of the proposed Bidirectional LSTM + Hyperopt model, (**a**) The training and validation loss, and (**b**) The training and validation accuracy for Dataset-1.

**Figure 10:**
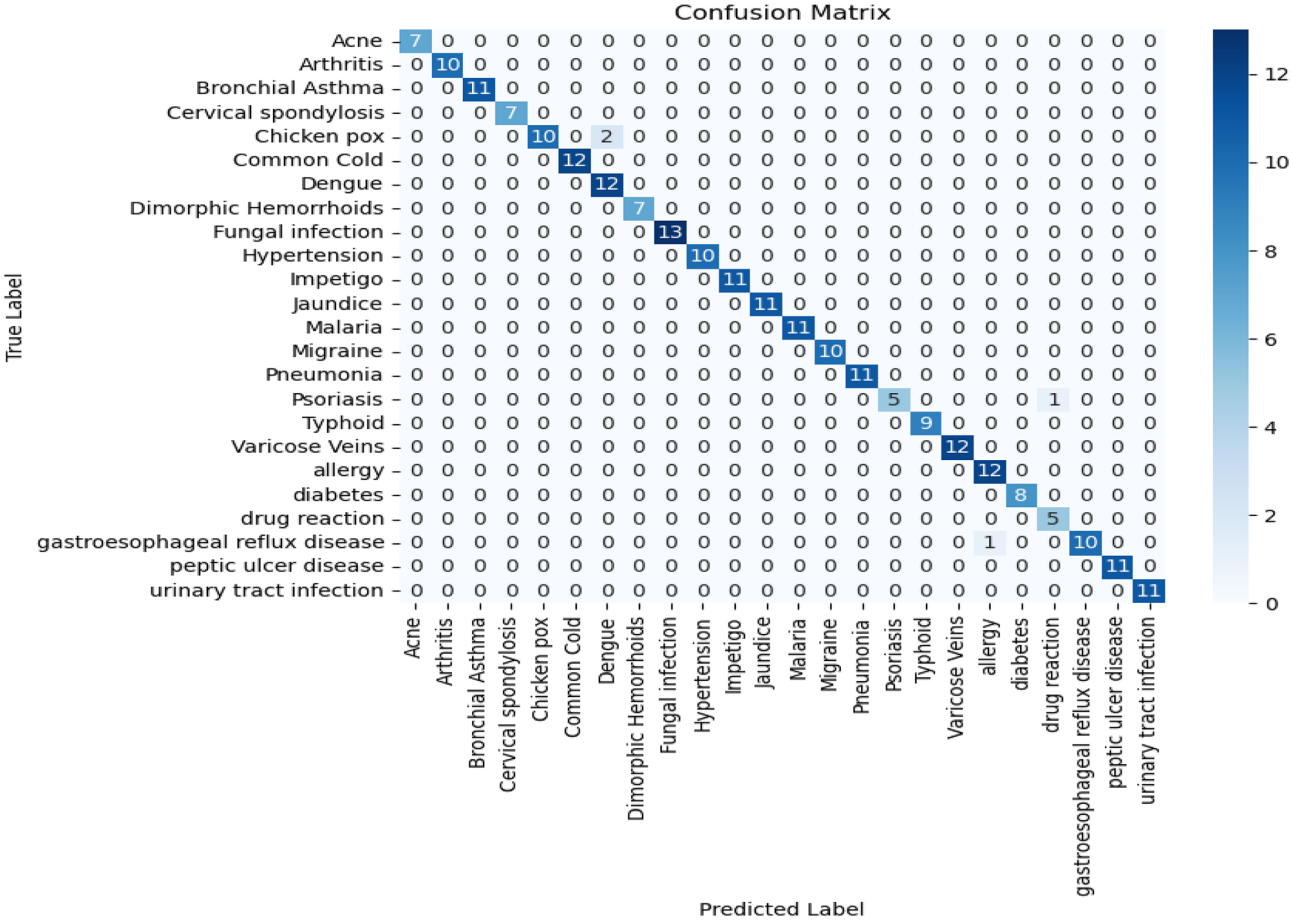
The confusion matrix for disease prediction for Dataset-1.

**Figure 11:**
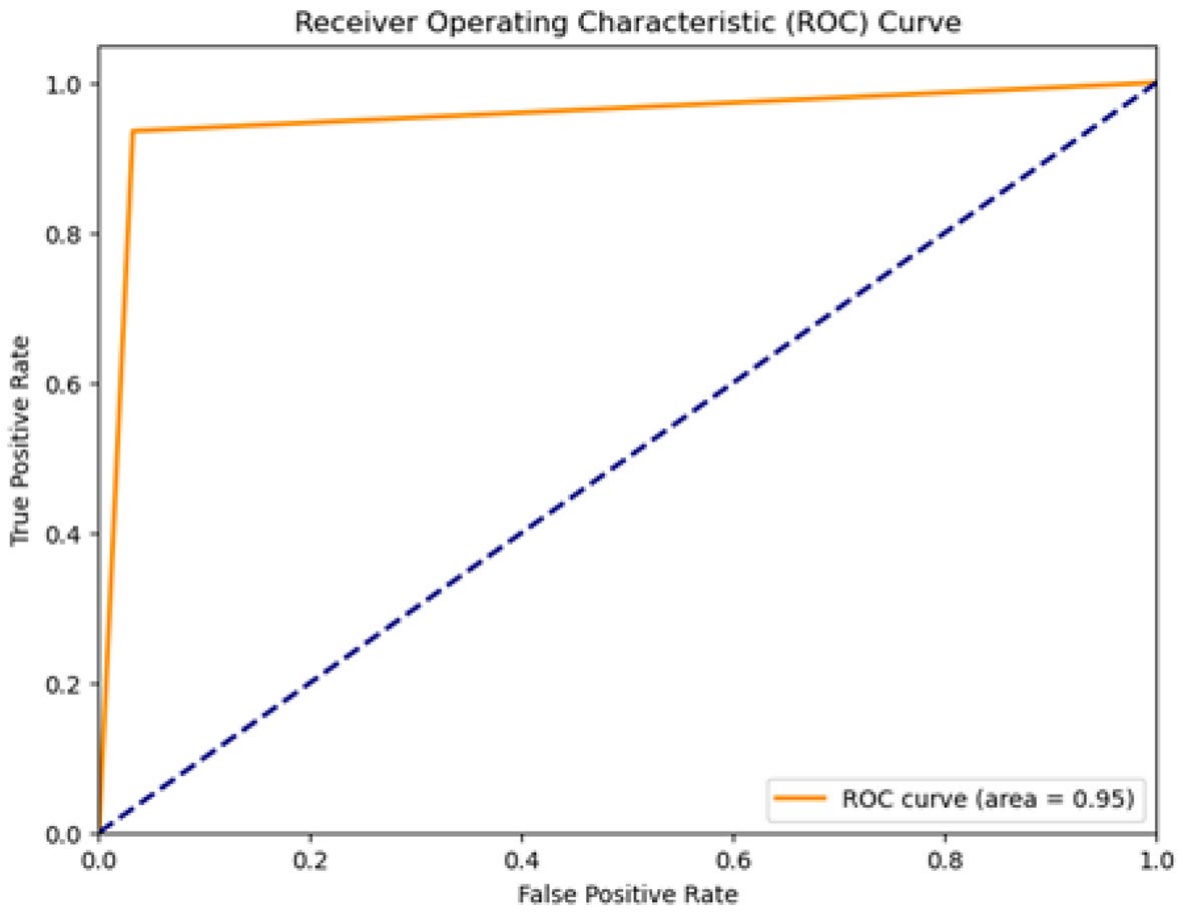
The ROC Curve for disease prediction for Dataset-2.

**Figure 12:**
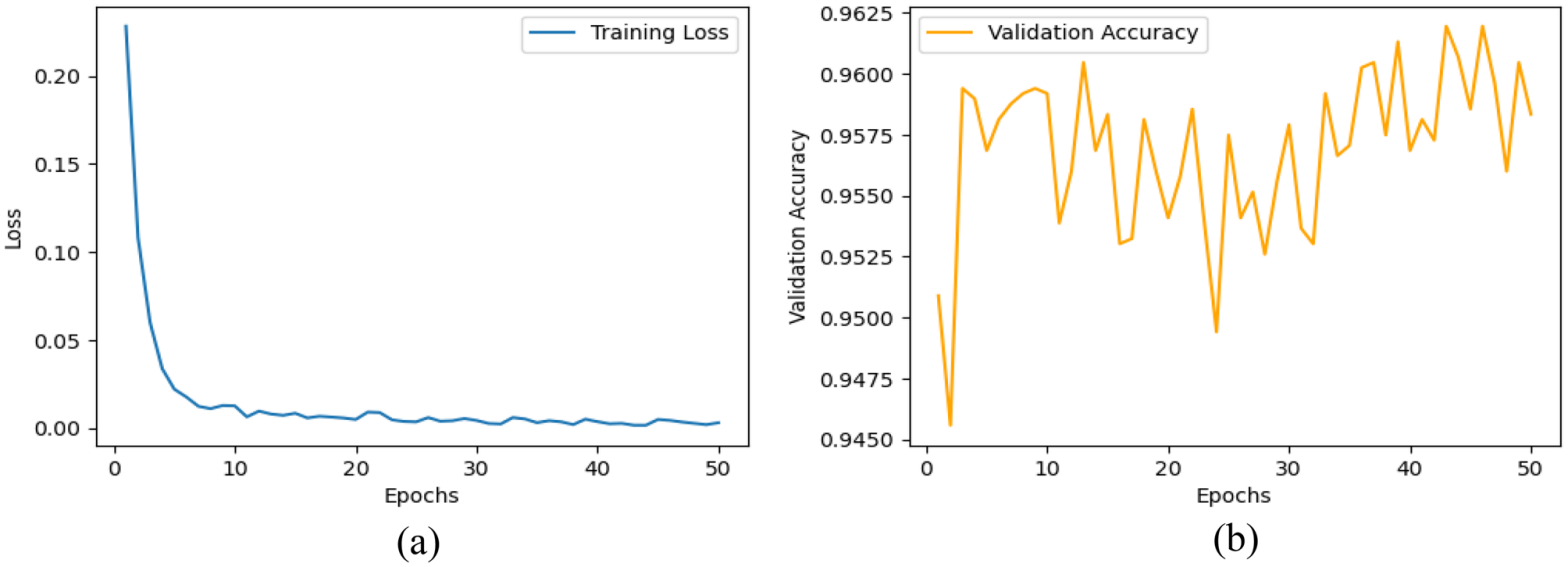
The learning Curve for twitter drug dataset for Dataset-2. (**a**) The training loss, and (**b**) The validation accuracy for Dataset-2.

We proposed the MCN-BERT model trained using multi-task learning on a medical text classification dataset (Dataset-1). The data was preprocessed by tokenizing the texts using the BERT tokenizer. We initialized MCN-BERT using the pre-trained BERT weights and fine-tuned it on Dataset-1. Two optimizers were evaluated—AdamP and AdamW. Figures 7 and 8 compare the training performance of MCN-BERT with the two optimizers. Each figure contains two subplots: (a) shows the training loss convergence, and (b) the validation accuracy assessing generalization. AdamP converged faster with lower training loss but AdamW achieved higher maximum accuracy. We then applied MCN-BERT to a disease classification task on Dataset-2. A bidirectional LSTM classifier with Hyperopt hyperparameter tuning was used. Figure 9 shows the training and validation loss/accuracy. Figure 10 is the confusion matrix evaluating classification performance. It displays the actual vs predicted disease labels, with cells indicating sample counts for each prediction. We computed classification metrics like accuracy, precision, recall and F1 score to assess overall and class-level performance. The ROC curve in Fig. 11 evaluates Binary disease classification on Dataset-2. Area under the ROC quantifies ability to distinguish presence/absence. Figure 12 presents the training and validation loss/accuracy curves for Dataset-2, indicating MCN-BERT generalizes well on this task.

The analysis of the results from Tables 4, 5 and 6:

- Across both datasets, MCN-BERT + AdamP consistently achieves the highest performance in terms of accuracy, F1 score, recall and precision compared to other models. This suggests it is the most effective approach for clinical named entity recognition.
- MCN-BERT + AdamW generally performs second best, indicating incorporating task-specific word embeddings like AdamW also improves upon the baseline MCN-BERT.
- BiLSTM + Hyperopt delivers lower but still strong performance, showing hybrid deep learning architectures can work well with hyperparameter optimization.
- Training times increase significantly for larger Dataset 2, as expected due to more training examples.
- Compared to prior work on Dataset 2, MCN-BERT + AdamP outperforms or matches SOTA models of Nguyen et al. and Hazell and Shakir according to available metrics.
- Only Chaichulee et al. achieve a slightly higher F1 score despite providing fewer performance metrics.

The proposed MCN-BERT models, particularly with AdamP, demonstrate state-of-the-art performance in clinical NER, highlighting the benefits of domain-specific pre-training and hyperparameter selection.

The performance of deep learning models depends heavily on selecting optimal hyperparameters. Tables 7 and 8 detail the hyperparameters optimized in this study for clinical named entity recognition using MCN-BERT and BiLSTM architectures, respectively. For MCN-BERT models, standard hyperparameters like batch size, epochs, learning rate, and device were applied based on best practices in the BERT literature. The Transformer-based model used the pretrained BERT layers with a classification head. Optimizers AdamP and AdamW were employed to fine-tune the model layers. Meanwhile, a broader hyperparameter search space was defined for the BiLSTM model to leverage the Hyperopt optimization algorithm. This included varied dimensions for the embedding layer, LSTM units, dropout rate, learning rate, and optimizer. Hyperopt efficiently searched this space to identify top-performing values for key recurrent network parameters.Specifying these hyperparameters systematically enabled fair comparisons between the deep learning approaches. Their selection based on prior work and automated tuning aimed to produce the best-performing configurations of each model for clinical data.

**Table 7:** The hyperparameters values of BERT.

Hyperparameter	Value
batch_size	32
Epochs	50
learning_rate	2e-5
num_labels	len(label_encoder.classes_)
max_length	128
Device	'cuda' if torch.cuda.is_available() else 'cpu'
Layer BERT	BertForSequenceClassification
Optimizer AdamP, AdamW	model.parameters(), lr = learning_rate, betas = (0.9, 0.999), weight_decay = 1e-6

**Table 8:** The hyperparameters values of Bi-LSTM + Hyperopt.

Hyperparameter	Values
embedding_dim	50, 100
lstm_units	64, 128
dropout_rate	Uniform distribution [0.2, 0.5]
learning_rate	Log-uniform distribution [0.00001, 0.1]
Optimizer	Adam
Hidden layers	1 Bidirectional LSTM layer
Loss function	Sparse categorical crossentropy
Batch size	16
Epochs	50

### Comparative study

In 14 studies, symptom-related information emerged as a key focus presented by Koleck et al.^38^. Electronic Health Record (EHR) narratives spanned various clinical specialties, including general, cardiology, and mental health, with general occurrences being the most frequent. The symptoms covered in these studies were diverse, encompassing issues such as shortness of breath, pain, nausea, dizziness, disturbed sleep, constipation, and depressed mood. The Natural Language Processing (NLP) approaches employed comprised previously developed tools, classification methods, and manually curated rule-based processing. However, only one-third of the studies (n = 9) provided information on patient demographic characteristics. The strategies used in these studies involved combinations of existing NLP tools, classification methods, and manually curated rule-based processing. Among the pre-existing NLP tools, the Medical Language Extraction and Encoding system and Text Analysis System were utilized. In terms of performance, the NLP system using SNOMED–CT for extraction demonstrated a sensitivity of 0.62 and specificity of 0.63 for any chest pain, sensitivity of 0.71 and specificity of 0.60 for exertional chest pain, and sensitivity of 0.88 and specificity of 0.58 for definitive Rose angina.

Putra et al.^26^ proposed a NLP system to enhance the Clinical Decision Support (CDS) process by identifying symptoms associated with digestive diseases. Named Entity Recognition (NER) was employed as the methodology to discern tokens indicative of the disease's symptoms. The model, trained over 50 epochs, achieved an f1-score accuracy of 0.79. Experimental findings indicate that incorporating stemming and the removal of stopwords in the pre-processing stage enhances the accuracy of the system.

Yu^39^ introduced a data mining framework focused on symptoms and diseases, aiming to construct a semantic linked knowledge graph for prevalent health conditions. The study demonstrated the capacity of machines to possess self-learning capabilities through a predefined knowledge graph schema, leveraging data retrieval from the web. Currently, they have generated 22,431 triple links associated with 212 health conditions, establishing relationships between symptoms and diseases, as well as between different diseases. These triples serve as valuable inputs for causal reasoning techniques, aiding in the filtration of potential diseases. A thorough literature search spanning 1964 articles from PubMed and EMBASE was meticulously narrowed down to 21 eligible articles. Pertinent data, encompassing the purpose of the studies, text sources utilized, the number of users and/or posts involved, evaluation metrics employed, and quality indicators, were systematically documented by Dreisbach et al.^40^. The clinical content categories most frequently under evaluation were pain (n = 18) and fatigue and sleep disturbance (n = 18). The studies accessed electronic Patient-Reported Outcome (ePAT) data from diverse sources such as Twitter, online community forums, or patient portals, with a focus on diseases including diabetes, cancer, and depression. Notably, 15 studies prominently featured Natural Language Processing (NLP) as a primary methodology. Evaluation metrics reported across studies included precision, recall, and F-measure, particularly for addressing symptom-specific research questions. A chatbot service, designated for the Covenant University Doctor (CUDoctor) telehealth system, has been crafted employing fuzzy logic rules and fuzzy inference presented by Omoregbe et al., 2020^41^. This specialized service is designed to evaluate symptoms associated with tropical diseases prevalent in Nigeria. The Telegram Bot Application Programming Interface (API) establishes the connection between the chatbot and the system, while the Twilio API facilitates connectivity between the system and Short Messaging Service (SMS) subscribers. The service draws upon a knowledge base enriched with established facts about diseases and symptoms derived from medical ontologies. To predict diseases effectively based on inputted symptoms, a fuzzy support vector machine (SVM) is employed. The user inputs are recognized through Natural Language Processing (NLP) and conveyed to CUDoctor for decision support. Subsequently, a notification message signaling the completion of the diagnosis process is dispatched to the user. The outcome is a medical diagnosis system offering personalized diagnostic insights, utilizing self-input from users for effective disease identification. To gauge the system's usability, an evaluation was conducted using the System Usability Scale (SUS), yielding a mean SUS score of 80.4. This score indicates an overall positive evaluation, affirming the efficacy and user-friendly nature of the developed system.

Koleck et al.^42^ presented synonym lists for each pilot symptom concept using the Unified Medical Language System. Subsequently, they leveraged two extensive text sources, comprising 5,483,777 clinical notes from Columbia University Irving Medical Center and 94,017 PubMed abstracts with Medical Subject Headings or relevant keywords related to the pilot symptoms, to further enrich their initial pool of synonyms for each symptom concept. For these tasks, they employed NimbleMiner, an open-source Natural Language Processing (NLP) tool. To assess the performance of NimbleMiner in symptom identification, they compared its results to a manually annotated set of 449 nurse- and physician-authored common Electronic Health Record (EHR) note types. In comparison to the baseline Unified Medical Language System synonym lists, their approach revealed up to 11 times more additional synonym words or expressions, including abbreviations, misspellings, and unique multi-word combinations, for each symptom concept. The NLP system demonstrated outstanding symptom identification performance, with F-measure scores ranging from 0.80 to 0.96. In the realm of user-generated text, particularly on platforms like social media and online forums, individuals often employ disease or symptom terms for purposes beyond describing their health status. The health mention classification (HMC) task in data-driven public health surveillance endeavors to distinguish posts where users discuss health conditions from instances where disease and symptom terms are used for other reasons. Current computational research primarily focuses on health mentions in Twitter, exhibiting limited coverage of disease or symptom terms and neglecting user behavior information and alternative uses of such terms. To propel HMC research forward, Naseem et al.^43^ introduces the Reddit Health Mention Dataset (RHMD), a novel dataset derived from multi-domain Reddit data designed for HMC. RHMD comprises 10,015 manually labeled Reddit posts referencing 15 common disease or symptom terms, categorized into four labels: personal health mentions, non-personal health mentions, figurative health mentions, and hyperbolic health mentions. Leveraging RHMD, we propose HMCNET, a methodology that integrates target keyword identification (disease or symptom term) and user behavior hierarchically to enhance HMC. Experimental results showcase that our approach surpasses state-of-the-art methods, achieving an F1-Score of 0.75, marking an 11% improvement over existing methodologies. Additionally, our new dataset, RHMD, presents a robust challenge to current HMC methods. Two distinct academic memory clinic cohorts, comprising the Amsterdam UMC cohort (n = 3001) and the Erasmus MC cohort (n = 646), were utilized in the study presented by Eikelboom et al.^44^. The patient pool in these cohorts encompassed individuals with Mild Cognitive Impairment (MCI), Alzheimer's Disease (AD) dementia, or mixed AD/Vascular Dementia (VaD). A total of ten trained clinicians annotated 13 types of Neuropsychiatric Symptoms (NPS) in a randomly selected training set of n = 500 Electronic Health Records (EHRs) from the Amsterdam UMC cohort and a test set of n = 250 EHRs from the Erasmus MC cohort. For each NPS, a generalized linear classifier was trained and subjected to internal and external validation. Prevalence estimates of NPS were adjusted to account for the imperfect sensitivity and specificity of each classifier. In a subsample (59%), an intra-individual comparison of the NPS classified in EHRs and NPS reported on the Neuropsychiatric Inventory (NPI) was conducted. Internal validation demonstrated excellent performance for the classifiers, with an Area Under the Curve (AUC) range of 0.81–0.91. However, external validation performance exhibited some variability, with an AUC range of 0.51–0.93. NPS were found to be prevalent in EHRs from the Amsterdam UMC cohort, particularly apathy (adjusted prevalence = 69.4%), anxiety (adjusted prevalence = 53.7%), aberrant motor behavior (adjusted prevalence = 47.5%), irritability (adjusted prevalence = 42.6%), and depression (adjusted prevalence = 38.5%). A similar ranking of NPS prevalence was observed for EHRs from the Erasmus MC cohort, although some classifiers faced challenges in obtaining valid prevalence estimates due to low specificity. In both cohorts, minimal agreement was identified between NPS classified in the EHRs and those reported on the NPI assessments, with all kappa coefficients being less than 0.28. Notably, there were considerably more reports of NPS in EHRs than in NPI assessments. Comparative study between the proposed model and the existing approaches is shown in Table 9.

**Table 9:** The comparative study between the recent approaches with the proposed model.

Author	Data used	Methodology	Results	Comments
Koleck et al., 2019^38^	Electronic Health Record (EHR) narratives	NLP approaches (previously developed tools, classification methods, and manually curated rule-based processing)	Various symptoms covered, NLP system performance reported	Patient demographic characteristics only provided in one-third of the studies
Putra et al., 2019^26^^2^	Not specified	Named Entity Recognition (NER)	NLP system achieved an f1-score accuracy of 0.79	Pre-processing enhancements improved system accuracy
Yu, 2019^39^	Not specified	Data mining framework, self-learning knowledge graph construction	Knowledge graph with 22,431 triple links generated	Graph used for causal reasoning techniques
Dreisbach et al., 2019^40^	PubMed and EMBASE articles	Literature search, NLP methods	Pain and fatigue/sleep disturbance were most frequently evaluated	NLP used in 15 studies, evaluation metrics included precision, recall, and F-measure
Koleck et al., 2021^42^	Clinical notes and PubMed abstracts	NimbleMiner NLP tool	NLP system achieved outstanding symptom identification performance	Improved synonym lists generated for symptom concepts
Naseem et al., 2022^43^	Reddit data	HMCNET methodology for health mention classification	RHMD dataset created, HMCNET outperformed existing methods	RHMD dataset poses a challenge to current HMC methods
Eikelboom et al., 2023^44^	Amsterdam UMC and Erasmus MC cohorts	Generalized linear classifiers, NPS prevalence estimation	Excellent performance in internal validation, variability in external validation	Prevalence estimates for various Neuropsychiatric Symptoms (NPS) reported
Proposed Model	Dataset1 and 2	MCN-BERT + AdamP	Accuracy: 96.15	MCN-BERT models designed for clinical Named Entity Recognition (NER), showcasing their superior performance. The models, particularly effective when coupled with the AdamP optimizer, surpass alternative approaches in the domain
		MCN-BERT + AdamW	Accuracy:95.15	
		Bidirectional LSTM + Hyperopt	Accuracy:94.15	

## Discussion

The study demonstrates the potential of leveraging advances in language models and deep learning for automating disease prediction from symptoms. The use of MCN-BERT models, optimized with AdamP and AdamW optimizers, and a BiLSTM model, optimized with Hyperopt, resulted in strong performance in predicting diseases from symptom descriptions. The MCN-BERT model with AdamP optimizer achieved the best performance, with an accuracy of 99.58%, F1 score of 99.13%, recall of 99.28%, and precision of 99.18%, while the MCN-BERT model with AdamW optimizer performed well with an accuracy of 98.33%, F1 score of 98.18%, recall of 98.23%, and precision of 98.39%. The BiLSTM model achieved an accuracy of 97.08%, F1 score of 97.05%, recall of 97.08%, and precision of 97.37%. The results of the study demonstrate the potential of language models and hyperparameter optimization for accurately predicting diseases from symptoms.

The use of MCN-BERT models and BiLSTM model showed promising results, with strong performance in predicting diseases from symptom descriptions. The study also highlights the importance of hyperparameter optimization in improving the performance of language models for disease prediction. The study has several implications for healthcare. Firstly, the use of language models and deep learning for disease prediction has the potential to revolutionize healthcare by supporting earlier detection, more prompt treatment, and expanding remote diagnostic capabilities. This could lead to improved patient outcomes and better management of diseases. Secondly, the study demonstrates the potential of automating disease prediction from symptoms, which could reduce the workload of healthcare professionals and improve the efficiency of healthcare systems. The study highlights the need for further research in this area, including the exploration of other models and approaches, to fully realize the potential of language models and deep learning for disease prediction. The results not only emphasize how tweaking certain parameters significantly boosts the language model's ability to predict diseases based on symptoms but also suggest significant potential benefits for the healthcare sector. The idea that healthcare could undergo a transformation, marked by earlier detection, quicker treatment, and more extensive remote diagnostic capabilities, is promising for achieving better patient outcomes and more effective disease management. The study also points out the potential for increased efficiency by automating disease prediction from symptoms, which could reduce the workload for healthcare professionals and improve overall healthcare system efficiency. However, the study is aware of its limitations, particularly the fact that the dataset is limited to a specific population. This limitation prompts considerations about how well the findings might apply to broader populations or different clinical settings, emphasizing the need for more exploration and validation. However, there are also some limitations to the study that need to be addressed in future research. The dataset used in the study was limited to a specific population and may not generalize to other populations or clinical settings.

The study recognizes the importance of interpretability in medical applications and emphasizes ethical considerations, particularly addressing biases in data and their potential impact on model predictions. By addressing these ethical concerns, the research aims to contribute to the development of reliable and ethically sound language models for disease prediction, ultimately improving patient care and outcomes.

The interpretability of the developed language models, MCN-BERT + AdamP and MCN-BERT + AdamW architectures, is crucial in the context of medical applications for clinical decision-making. The ability to understand and interpret the decisions made by these models is essential for healthcare professionals to trust the predictions and integrate them into the diagnostic process. Interpretability ensures transparency in the model's decision-making process, allowing clinicians to comprehend how specific symptoms contribute to disease predictions. This transparency is vital for building confidence in the model's recommendations and fostering collaboration between the automated system and medical practitioners.

The ethical considerations of the current study includes:

1. *Biases in data* The study acknowledges the potential biases present in medical data, emphasizing the importance of mitigating these biases. Biased training data can lead to unfair or inaccurate predictions, disproportionately affecting certain patient demographics. The research should explicitly detail strategies employed to identify, understand, and address biases in the training data, ensuring that the models deliver equitable and unbiased predictions across diverse patient populations.
2. *Impact of biases on predictions* An ethical consideration involves a thorough exploration of how biases in the data might impact model predictions. The study should address the potential consequences of biased predictions on different patient groups, emphasizing the need for fairness and equity in disease predictions. Transparent reporting on potential disparities ensures that healthcare professionals are aware of the limitations and potential ethical implications associated with the model's outputs.
3. *Model transparency and accountability* The research should explicitly discuss measures taken to enhance model transparency, making the decision-making process interpretable for clinicians. Ensuring accountability in the model's predictions is essential for ethical deployment in real-world healthcare scenarios. By providing a clear understanding of the model's inner workings, the study contributes to ethical AI practices in healthcare.
4. *Real-world validation* Ethical considerations should extend to the validation of the models in real-world healthcare settings. The study should discuss plans for evaluating the models' performance in diverse clinical scenarios, addressing the challenges and ethical implications of implementing these models in actual patient care. Real-world validation ensures that the models align with ethical standards and demonstrate effectiveness in practical healthcare applications.

## Limitations

Despite the promising results of the study, there are several limitations that need to be addressed in future research:

- *Data quality and representation* The dataset used in the study was limited to a specific population and may not be generalizable to other populations or clinical settings. The dataset also relied on self-reported symptoms, which may be subject to biases and inaccuracies.
- *Model interpretability* The study used complex machine learning models, such as MCN-BERT and BiLSTM, which can be difficult to interpret and understand. This lack of interpretability may limit the clinical usefulness of the models, as healthcare professionals may struggle to understand the reasoning behind the predictions.
- *Training time* The study found that training the MCN-BERT models with AdamP optimizer and AdamW optimizer took significant amounts of time, which may be a limitation for clinical settings where fast and accurate predictions are crucial.
- *Limited domain knowledge* The study focused on a limited number of diseases and symptoms, which may not capture the full range of diseases and symptoms that can occur in clinical practice.
- *Lack of domain expertise* The study did not involve domain experts in the field of medicine, which may have limited the understanding of the clinical relevance of the predictions and the accuracy of the models.
- *Limited testing* The study did not perform extensive testing to evaluate the performance of the models in different clinical settings and populations, which may limit the generalizability of the findings.
- *Lack of integration with clinical systems* The study did not explore the integration of the language models with clinical systems, such as electronic health records or clinical decision support systems, which may limit their utility in real-world clinical settings.
- *Need for further research* The study highlights the promise of language models and hyperparameter optimization for accurately predicting diseases from symptoms, but further research is needed to overcome the limitations of the study and to explore the full potential of this approach.

## Conclusion and future work

This study investigated the use of state-of-the-art natural language processing and deep learning techniques for clinical named entity recognition from electronic health records and biomedical literature. Specifically, we compared two MCN-BERT models optimized with AdamP and AdamW against a BiLSTM model tuned with Hyperopt. Using two largebenchmark datasets, we aimed to automatically identify and classify medical entities like diseases, symptoms and adverse drug reactions from unstructured text. The experimental results demonstrate that the MCN-BERT approach optimized with AdamP achieved the best performance overall, attaining accuracies of 99.58% and 96.15% on Datasets 1 and 2 respectively. The MCN-BERT model with AdamW optimization also delivered strong results, outperforming the BiLSTM baseline. Overall, our proposed domain-adapted transformer architectures yielded superior clinical named entity recognition compared to prior work. These findings have important implications. By effectively extracting structured information from unstructured notes, clinical language models can support clinical decision making, drug safety surveillance, and knowledge discovery. Automatic identification of diseases and adverse events also paves the way for improved computational Phenotyping and pharmacovigilance. Looking ahead, further advances in model architectures and leveraging larger healthcare datasets hold promise to advance the state-of-the-art in medical natural language processing. The accuracy levels observed also suggest clinical language models are reaching maturity for real-world applications. Overall, our study underscores the growing potential of artificial intelligence to transform healthcare by unlocking insights from the tremendous amounts of textual patient data. This study demonstrated promising results for clinical named entity recognition using MCN-BERT models, however future research is still needed to advance these techniques for real-world clinical applications. Larger and more diverse healthcare datasets could be utilized to validate the generalizability of these models, particularly on underrepresented patient populations. Incorporating additional context like demographics, medical history and temporal trends can improve performance by providing a more holistic view of the patient. Multi-task learning approaches that jointly solve related problems such as relationship extraction and coding assignment could generate a more comprehensive understanding compared to single-task models. Leveraging self-supervised pre-training strategies has the potential to make better use of unlabeled clinical data. Integrating these models into clinical decision support systems and evaluating their impact on downstream tasks from diagnosis to treatment planning would help establish their clinical value. In the future work, we further plan to use another recent predictors such as pAtbP-EnC^45^, AIPs-SnTCN^46^, AFP-CMBPred^47^, cACP-DeepGram^48^, iACP-GAEnsC^49^, and Target-ensC_NP. Furthermore, we intended to used the CD-HIT tool was utilized to eliminate redundant peptide samples with homology^50^.

Continued development of explainable AI is also important for gaining user trust in model-driven healthcare. Further optimizing model architectures and expanding available data sources holds promise to consolidate medical language processing as a key enabling technology for advancing precision medicine through insights from patient narratives.

## Data Availability

The data that support the findings of this study are available as follows. Dataset 1: https://www.kaggle.com/datasets/niyarrbarman/symptom2disease/. Dataset 2: https://data.mendeley.com/datasets/f7mrczj83k/1. Source of Dataset-2: https://www.kaggle.com/datasets/pawan2905/tweet-classification?select=Data.csv.
